# Experimental observations of communication in blackout, topological waveguiding and Dirac zero-index property in plasma sheath

**DOI:** 10.1515/nanoph-2022-0800

**Published:** 2023-04-11

**Authors:** Jianfei Li, Ying Wang, Zhongxiang Zhou, Jingfeng Yao, Jianlong Liu, Zhihao Lan, Chengxun Yuan

**Affiliations:** School of Physics, Harbin Institute of Technology, Harbin 150000, People’s Republic of China; Heilongjiang Provincial Key Laboratory of Plasma Physics and Application Technology, Harbin 150000, People’s Republic of China; Key Laboratory on In-Fiber Integrated Optics of Ministry of Education, College of Physics and Optoelectronic Engineering, Harbin Engineering University, Harbin 150001, China; Department of Electronic and Electrical Engineering, University College London, Torrington Place, London WC1E 7JE, UK

**Keywords:** communication blackout, Dirac cone, evanescent waves, plasma photonic crystal, topological edge states

## Abstract

The plasma sheath causes the spacecraft’s communication signal to attenuate dramatically during the re-entry period, which seriously threatens the astronauts. However, valid experimental protocols have not been obtained hitherto. To realize the propagation of electromagnetic waves in negative permittivity background of the plasma sheath, alumina columns are embedded in the plasma background to form plasma photonic crystals, which can support the coupling of evanescent waves between the alumina columns. We experimentally demonstrate the realization of communication in blackout scenario by achieving a complete passing band in the plasma cutoff region. For high frequency communications in the plasma sheath, electromagnetic wave propagation based on topological edge states is also experimentally demonstrated. Furthermore, we realize a triply-degenerate Dirac cone formed dynamically at the center of the Brillouin zone by modulating the electron density, where electromagnetic wave exhibits high transmittance and does not experience phase accumulation at the Dirac point. Our work thus not only provides an effective approach to overcome the communication blackout problem, but the design can also be served as a promising experimental platform to explore topological electromagnetic phenomena.

## 1 Introduction

The problem of communication blackout is of great importance due to the loss of electromagnetic signals between the spacecraft and the ground, which lasts for up to ten minutes. It originates from the plasma sheath formed by the friction between high-speed spacecraft and the surrounding atmosphere during reentry process, where the generated plasma frequency by the aircraft at speed of 15 Mach is in the range of 1–10 GHz and the thickness of the plasma layer is about 10 cm [1, 2]. When the incident frequency of electromagnetic waves is lower than the plasma frequency, the plasma will serve as a single negative-refractive-index material and as such, electromagnetic waves decay inside the plasma in the form of evanescent waves. Communication blackout has puzzled the aerospace industry for decades, and there is no satisfactory solution up to now. Considerable efforts have been proposed to alleviate the effect of plasma sheath by injecting electrophilic chemicals, adding magnetic windows and increasing the frequency of the incident wave [3, 4]. However, such intuitive methods of reducing electron density are difficult and energy-intensive to implement, and they only mitigate the effects of plasma on electromagnetic waves rather than act as fundamental solutions. In addition to the ionized gas (plasma) heated by high temperature, impurities with large particle sizes are produced from the ablation of protective layer [5]. High-frequency communication can overcome the plasma cutoff effect in the *Ka* or terahertz band [6, 7], but electromagnetic waves will be strongly scattered by impurities, resulting in severe amplitude and phase jitter of the signal. The reduction of signal quality will cause the acquisition and tracking of the receiving system to disappear, which leads to consequences similar to blackout [8, 9]. For high-frequency communication, it is the high expectation for robust propagation of electromagnetic waves in the paths containing impurities. Based on optical-mechanical analogy, it was theoretically proposed that a dielectric layer with extremely large permittivity could be embedded in the interval between spacecraft and plasma to amplify electromagnetic waves [10]. This method creatively introduces the concept of particle resonant tunneling through potential barriers, and thus provides a new idea to solve the communication blackout problem. After that, a theoretical approach based on metamaterial was proposed to enable wireless communication through a plasma sheath, where split-ring resonators supporting negative permeability are inserted into the plasma background (the plasma permittivity is negative at the specific frequencies), and a double-negative medium is formed to support transmission of electromagnetic waves [11]. So, experimentally and theoretically feasible methods are urgently required to realize electromagnetic signal propagation in the plasma sheath.

The proposal of photonic crystals (photonic analogues to electronic semiconductors) has provided new ways for manipulating the propagation of electromagnetic waves. Novel concepts in the field of photonic crystals have been explored in-depth and many significant achievements have been made, such as symmetry-protected scattering anomaly [12], topological edge state [13–15], and zero-refractive-index property based on Dirac-like cones [16, 17]. In the study of photonic crystals, tight-binding theory in analogy with electron systems is a common method [18, 19]. However, there is a crucial difference that while electromagnetic waves can propagate in the air, the electron wave function decays rapidly in the background and evanescent waves coupled between atoms in electronic systems could be described by tight-binding models [20]. To mimic the damping of electromagnetic waves, it was theoretically proposed that metals could be inserted into the air background to form photonic crystals, which can produce surface plasmon modes on the metal surfaces [21]. Similarly, metallic photonic crystals with three-dimensional cubic lattices were also designed to achieve tight-binding photonic bands, where the photonic bound states are localized around the crossing of the metallic lattices [22]. In the communication blackout problem, the plasma sheath around the spacecraft provides a negative-permittivity background for the propagation of electromagnetic waves. In this work, two ideas are proposed and experimentally verified for addressing the blackout communication problem. One is the use of tight-binding photonic bands to achieve electromagnetic wave propagation in the negative-permittivity background of the plasma. The other is to improve the signal quality in the high frequency band by using topological waveguiding and Dirac zero refractive-index property.

## 2 Experimental setup and theoretical framework

**Figure 1:**
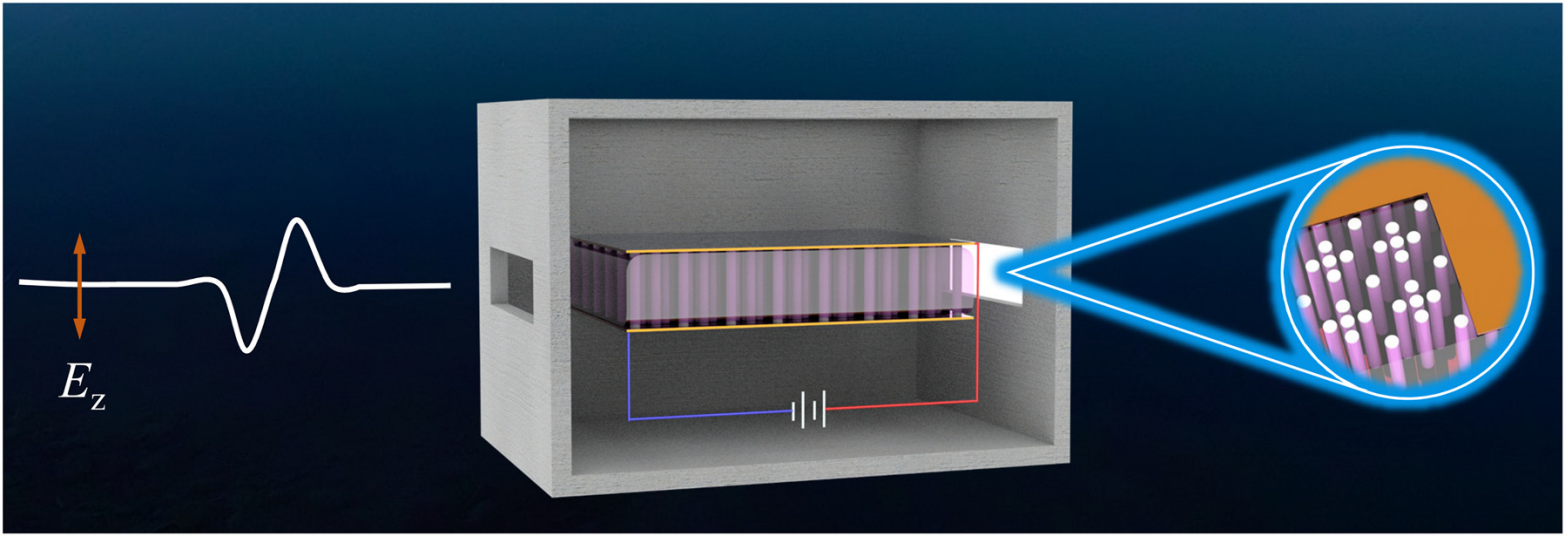
Schematic illustration of the experimental setup for implementing plasma photonic crystals, where alumina columns are embedded into a plasma background formed between two electrodes in a vacuum chamber.

The apparatus for studying electromagnetic wave propagation in plasma photonic crystals is schematically illustrated in Figure 1. In specific, two electrodes with a length of 12 cm and a width of 10 cm are placed parallel to each other in the vacuum chamber, maintaining 1.5 cm between the electrodes. Argon is injected into the chamber and the pressure in the chamber is controlled at 60–120 Pa by a vacuum pump system. Then, uniform plasma can be generated by a direct current power source, which has a length conforming to the plasma sheath around the spacecraft. In this work, we only consider the transverse magnetic (TM) polarization of the electromagnetic wave, i.e., *E*_*z*_. This mode satisfies the condition that the tangential component of the electric field at the electrode surface is zero, which makes the electrode act as a perfect electrical conductor. In experiments, planar metallic waveguide chamber could be used for TM modes [23, 24]. The electromagnetic wave is emitted from a horn and passes through the electrodes which serve as a waveguide under TM polarization. Next, alumina columns with permittivity of 9.4 are embedded into the plasma background to form plasma photonic crystals, which have interesting properties such as tunability and reconfigurability [25–27].

In the study of all-dielectric photonic crystals, the band structure can be calculated by solving the Hamiltonian eigenequation $\hat{\Theta }H={\left(\frac{\omega }{c}\right)}^{2}H$ , where $\hat{\Theta }$ is a linear operator denoted as $\nabla \times \frac{1}{\varepsilon \left(r\right)}\nabla \times$ . However, the permittivity ( $\varepsilon_{r}=1-\frac{\omega_{pe}^{2}}{\omega \left(\omega -i\nu \right)}$ , where *ω*_*pe*_ is the plasma frequency and *ν* is the collision frequency) of plasma background in this work is frequency-dependent, which can be derived from Drude model [28, 29]. So, the main equation is no longer a standard eigenvalue problem. For this case, auxiliary fields are usually introduced to transform the main equation to a standard matrix eigenvalue problem [21, 30]. To analyze the dispersive properties of the gaseous plasma background, the electron motion equation of plasma is employed, where gaseous plasma is generated at low pressure (60–120 Pa) and without a magnetic field. As such, the collision frequency of electrons with neutral particles, which is related to the gas pressure, is neglected [31]. For the reentry plasma, a temperature of 2000 K results in a collision frequency of 42.5 MHz, which is much lower than the plasma frequency [32]. The effect of the dispersive medium actually modifies the eigenmode, which is related to the energy density of the electromagnetic wave as follows (see Appendix A for details),

(1) $$\begin{array}{ll} \int dry_{m}\cdot y_{n} & =\int dr\left[\mu_{0}H_{m}^{*}\cdot H_{n}+\varepsilon_{0}\left(1-\frac{\omega_{pe}^{2}}{\omega_{m}\omega_{n}}\right)E_{m}^{*}\cdot \;E_{n}\right] \end{array}$$

where **y**_*m*_ and **y**_*n*_ are the eigenmodes with eigenfrequency of *ω*_*m*_ and *ω*_*n*_, respectively.

## 3 Results and discussion

### 3.1 Realization of a complete passing band within the plasma cutoff region

**Figure 2:**
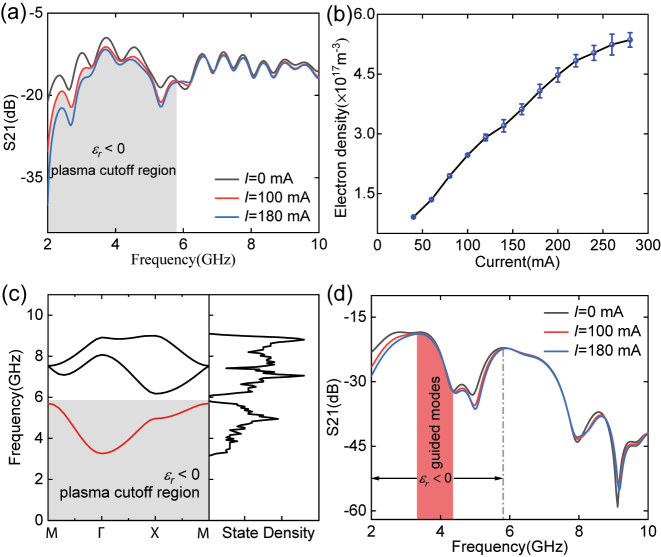
Experimental demonstration of communication in blackout. (a) Transmission of electromagnetic waves in uniform plasma at different discharge currents. (b) Electron density of the plasma measured by the wave propagation method. (c) Calculated band structure of the plasma photonic crystal when alumina columns are embedded into the plasma, where a complete passing band appears within the plasma cutoff region. (d) Transmission of electromagnetic waves in the plasma photonic crystal at different discharge currents. A window (red shaded region) can be clearly identified where the transmission does not change at different discharge currents due to the existence of guided modes in the plasma cutoff region.

When the pressure in the chamber is 60 Pa, the gas will breakdown by the applied electric field between the electrodes, and the gaseous plasma is generated. According to the permittivity of plasma, the cutoff effect occurs when the frequency of the electromagnetic wave is less than the plasma frequency. Figure 2(a) shows the result of the TM wave propagation in the plasma. It is obvious that the transmittance decreases with the increase of discharge current below 5.8 GHz, where the plasma cutoff region appears. We use the wave propagation method to diagnose the plasma electron density [33, 34], where electromagnetic waves with frequencies above 30 GHz are sent to pass through the plasma, which allows the electromagnetic waves to propagate through the plasma for several cycles. The average electron density at different currents is obtained by the phase difference according to Equation $\Delta \phi =\frac{k_{0}e^{2}}{2\varepsilon_{0}m\omega^{2}}\int_{0}^{l}n\left(x\right)dx$ , where *k*_0_ is the propagation constant in vacuum, *e* the unit charge, *m* electron mass, *ω* the frequency of incident wave, and *l* the plasma length. We assume that the plasma generated by plate electrode discharge is homogeneous, and the average electron densities at different currents are obtained as shown in Figure 2(b). The electron density increases linearly with the discharge current and its value is on the order of 10^17^ m^−3^. The experiment reproduces the propagation of electromagnetic waves in plasma sheath on the surface of the spacecraft.

To achieve electromagnetic wave propagation in the plasma cutoff region, we construct a lattice that contains six sites in the unit cell with a lattice constant of 20 mm (see Figure 1). Based on the measured electron density, the calculated band structure and density of states are shown in Figure 2(c). Due to the periodicity of the plasma photonic crystal, the band structure is calculated in a unit cell. Floquet conditions are applied to the boundaries, guaranteeing that the wave function varies by a phase factor within a period. We calculate the state density of the eigenmodes according to $N\left(\omega \right)=\sum_{n}\int_{BZ}d^{2}k\delta \left[\omega -\omega_{n}\left(k\right)\right]$ , where BZ is the first Brillouin zone, and $\omega_{n}\left(k\right)$ represents the eigenfrequency of the *n*th band [35]. From Figure 2(c), one can see that the first band (red line) is completely immersed within the plasma cutoff region, and an omnidirectional band gap appears between the first band and the second band, which can also be identified in the density of states. The measured transmission spectrum is shown in Figure 2(d), from which one can see that there are guided modes in the range of 3.3–4.4 GHz, and the modes are not affected by the discharge currents. However, the transmission on both sides of the guided modes is attenuated with the increase of the currents. The position of passing band can be easily tuned by changing the lattice structures or lattice constants (see Appendix B for demonstration). Therefore, we experimentally verify that the eigenfrequencies are consistent with the theoretical results. The design enables the transmission of electromagnetic in the plasma cutoff region.

### 3.2 Topological waveguiding in the plasma photonic crystal

**Figure 3:**
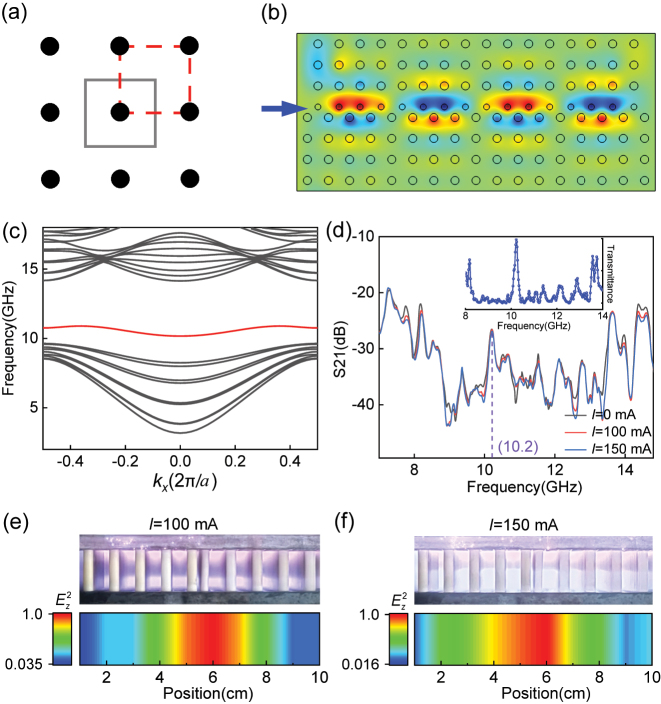
Topological waveguiding in the plasma photonic crystal. (a) Two different choices of the unit cell. (b) Lattice structure that supports a waveguide channel based on topological edge modes. (c) Projected band structure of the lattice in (b), where topological edge modes emerge within the bandgap. (d) Experimentally measured transmission of electromagnetic waves along the waveguide channel at different currents and the measured transmittance in the path containing impurities, where a clear peak corresponding to the topological edge mode can be identified. Electric field distribution at the output port of the waveguide with a discharge current of (e) 100 mA and (f) 150 mA.

Another idea to solve the communication blackout problem is to increase the frequency of the electromagnetic waves above the plasma cutoff frequency, where the relative permittivity of the plasma is positive and varies from 0 to 1. Thus the propagation of electromagnetic waves in this regime will not be blocked by the negative permittivity of the plasma background. Furthermore, the robust transmission properties of waveguiding via topological edge modes are suitable for making the electromagnetic waves impervious to perturbation by spatial impurities. To demonstrate this, we construct plasma photonic crystals by alumina columns in simple square lattices which are convenient to implement in practice. For a simple square lattice, there are two choices for the unit cell as shown in Figure 3(a). While the red unit cell has the nontrivial property, the gray unit cell is topologically trivial, which can be determined by the number of alumina columns *N* at one edge of the unit cell ( $N=\frac{N_{b}}{2}+\frac{N_{c}}{4}$ , where *N*_*b*_ and *N*_*c*_ represent the number of half-columns at the boundary and quarter-columns at the corner, respectively) [36, 37]. The different topological properties between the two choices of the unit cell ensure the presence of topological edge states according to the bulk-boundary correspondence. The plasma photonic crystal structure constructed by the two choices of the unit cell is shown in Figure 3(b), where we have used smaller columns at the interface rather than half-columns to construct the topological waveguide channel.

When alumina columns are embedded in the plasma background with electron density of 2.9 × 10^17^ m^−3^, the projected band structure of the plasma photonic crystal structure in Figure 3(b) is calculated and shown in Figure 3(c), where a topological edge state appears in the bandgap as shown by the red line. During calculation, the Floquet conditions are imposed on the *x*-direction boundaries of the superlattice, and the open conditions are applied on the *y*-direction boundaries. The wave vector is scanned only in the *x* direction to obtain the projection of the band structure. The measured transmission of electromagnetic waves along the waveguide channel at different discharge currents is shown in Figure 3(d). From the results, one can see that there is a wide bandgap between 8.2 and 13.5 GHz, which is consistent with the results of theoretical calculations in the Γ − *X* direction. In the bandgap, a distinct transmission peak can be identified at 10.2 GHz corresponding to the topological edge state and when the discharge current is changed from 100 mA to 150 mA, the transmission peak is not disturbed by the plasma. The topological edge state has robust properties that are immune to impurities or defects. When impurities are inserted into the transmission path and discharge current is controlled at 150 mA, the transmittance is shown in the inset of Figure 3(d). It is obvious that the electromagnetic wave still maintains a high transmittance at 10.2 GHz. However, the transmittance near 13.9 GHz is reduced due to the interference of impurities. To show the localization of the electric field at the interface, we scan the electric field intensity at the output port as shown in Figure 3(e) and (f). The high electric field energy is concentrated at the position of 6 cm corresponding to the interface, and the electric field distribution of the edge state is not changed by the plasma. Electromagnetic waves are strongly scattered as they propagate through impurity-contained plasma. Jitter in amplitude and phase degrades the signal quality of the receiving antenna. Thus, the topological edge states realized in this work can be well adopted to resist the interference of impurities.

### 3.3 Experimental realization of a tunable Dirac-like cone

**Figure 4:**
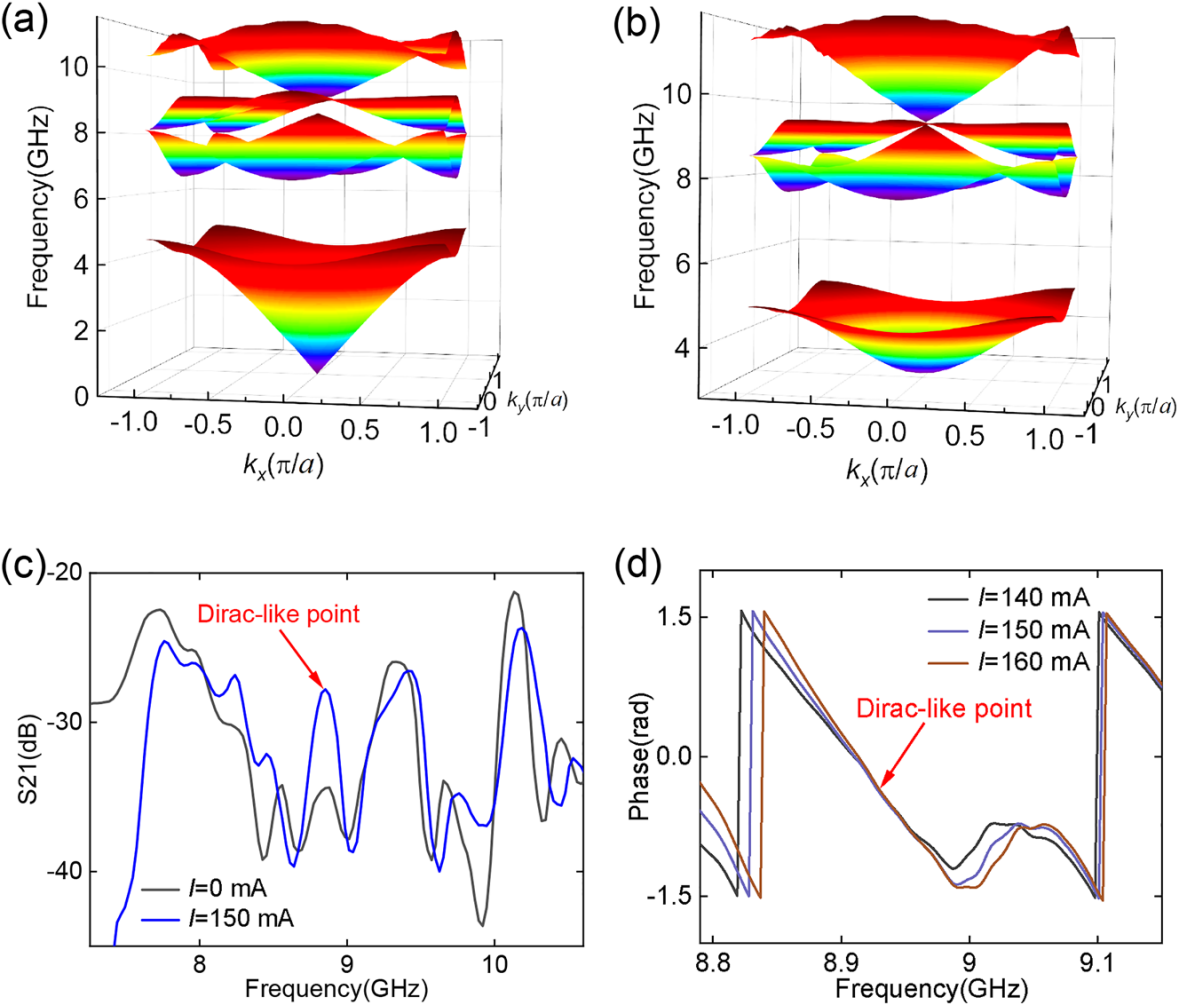
Experimental demonstration of Dirac zero-index property of the plasma photonic crystal. Calculated band structure of the plasma photonic crystal when the electron density is (a) zero and (b) 2.9 × 10^17^ m^−3^. (c) Experimentally measured transmission spectra with and without the discharge current, where a transmission peak corresponding to the Dirac point can be identified. (d) Experimentally measured phase variations at different discharge currents, where no phase accumulation occurs at the Dirac-like point.

A triply-degenerate point can be formed in the all-dielectric photonic crystals in square lattice by adjusting the lattice constant and dielectric permittivity, which is called accidental degeneracy [16, 17, 38]. A photonic crystal with a Dirac-like cone at the Γ point is an effective zero-refractive-index material with *ɛ*_eff_ = *μ*_eff_ = 0, which is often used in the study of object stealth [39–41]. However, this stealth effect is only valid for a single frequency, and the Dirac-like point cannot be tuned. It will be of great interest if the single frequency of the Dirac-like point is applied to communication. According to this theory, we construct a square lattice of alumina columns with radius of 4 mm and lattice constant of 20 mm. When the electron density is zero, the calculated band structure is shown in Figure 4(a), from which one can see that a bandgap is created between the second and third bands, while the third and fourth bands form a double degeneracy point. When the electron density is increased to 2.9 × 10^17^ m^−3^, a triply-degenerate Dirac-like cone is formed at Γ point as shown in Figure 4(b). Dirac-like cone in plasma photonic crystals also has the property of zero-refractive-index, which was confirmed theoretically in literature [42, 43]. We measure the electromagnetic wave transmission spectrum as shown in Figure 4(c). When plasma is not generated, the attenuation peak exists between 8.1 and 9.3 GHz corresponding to the bandgap between the second and third bands in Figure 4(a). The transmittance increases by 6.6 dB at 8.9 GHz when the discharge current increases to 150 mA, which corresponds to the Dirac-like point in Figure 4(b). To prove the validity of the experimental Dirac-like point, we measure the phase variation of S21 as shown in Figure 4(d). When the discharge current varies around 150 mA, electromagnetic wave does not experience any phase accumulation at Dirac-like point. For other frequencies deviating from the Dirac-like point, the overall phase shift occurs at different discharge currents. The incident wave has the zero-refractive-index property at the Dirac point, which can guide the waves around impurities. Instead, these impurities cause strong interference when electromagnetic waves propagate in the plasma. Therefore, the zero-refractive-index property of the Dirac-like cone will be applicable in communication between the reentry spacecraft and the ground.

## 4 Conclusions

In summary, we have investigated electromagnetic wave propagation in plasma photonic crystals with plasma properties mimicking the plasma sheath environment. First, we have experimentally achieved the transmission of electromagnetic waves in the plasma cutoff region by constructing complex lattices with alumina columns. For the high frequency communications in the plasma sheath, we have demonstrated topological waveguiding via topological edge states along a domain-wall interface between two plasma photonic crystals with different topological properties, where a transmission peak in the bandgap corresponding to the topological edge states can be identified. Furthermore, a Dirac-like cone in a square lattice is realized by modulating the electron density, which possesses the property of zero-refractive-index. The transmittance is increased by 6.6 dB and the phase does not accumulate at the Dirac-like point. Thus through a combination of simulations and experiments, our work demonstrates the great potential of manipulating electromagnetic wave propagation in plasma photonic crystals, which can not only effectively overcome the communication blackout problem, but the setup could also be used to explore topological physics in plasma.

## Figures and Tables

**Figure 5:**
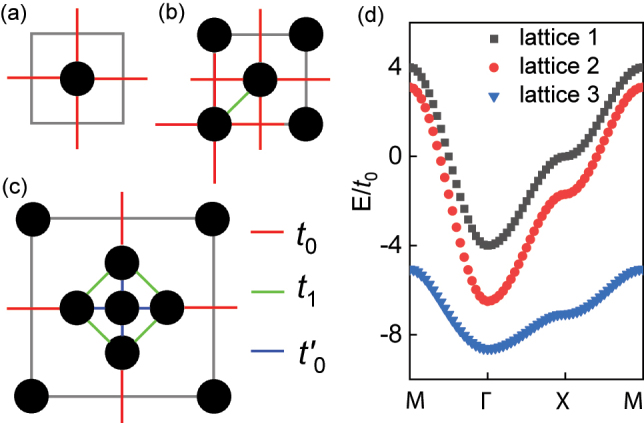
The description of tight-binding model. (a) The hopping pattern of lattice 1 which contains one site; (b) the hopping pattern of lattice 2 which contains two sites; (c) the hopping pattern of lattice 3 which contains six sites; and (d) the lowest energy bands for the three lattices at $t_{0}=1,t_{1}=1.4,t_{0}^{\prime }=2$ .

**Figure 6:**
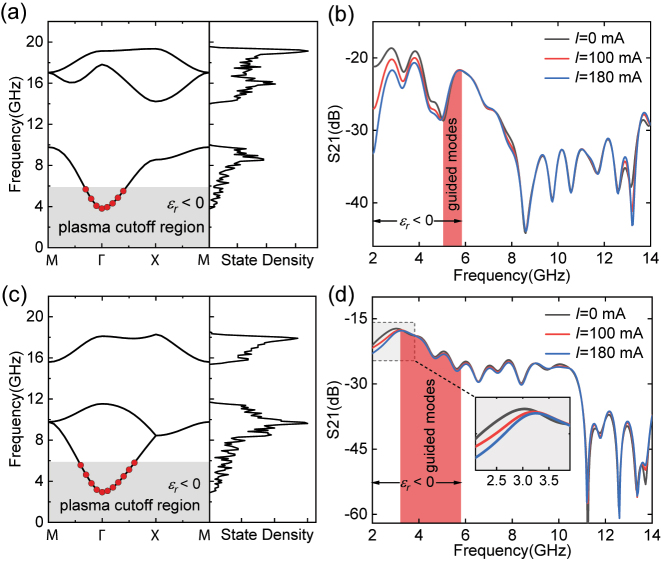
Theoretical and experimental results of lattices 1 and 2. (a) The calculated band structure using the measured electron density when one alumina column is embedded into the plasma. (b) Propagation of electromagnetic waves in the plasma photonic crystal in lattice 1 of Figure 5(a). (c) The calculated band structure using the measured electron density when two alumina columns are embedded into the plasma. (d) Propagation of electromagnetic waves in the plasma photonic crystal in lattice 2 of Figure 5(b).

## Appendix A: The matrix eigenequation containing collisionless plasma

We present the basic equations of the electromagnetic field as follows:

(A.1)
$$\frac{\partial H}{\partial t}=-\frac{1}{\mu_{0}}\nabla \times E$$

(A.2)
$$\frac{\partial E}{\partial t}=\frac{1}{\varepsilon_{0}}\nabla \times E-\frac{1}{\varepsilon_{0}}J$$

To analyze the dispersive properties of the gaseous plasma background, the electron motion equation of plasma is employed. Gaseous plasma is generated in low pressure (60–120 Pa) and without a magnetic field; hence, the collision frequency of electrons with neutral particles is neglected:

(A.3)
$$\frac{\partial J}{\partial t}=\varepsilon_{0}\omega_{pe}^{2}E$$

where **J** is the polarization current density, *ɛ*_0_ the permittivity in the vacuum, **E** the electric field, *ω*_pe_ the plasma frequency which is defined as $\omega_{pe}=\sqrt{ne^{2}/\varepsilon_{0}m_{e}}$ with *n* the electron density, *e* the unit charge and *m*_*e*_ the electron mass.

When all fields vary with exp(*iωt*), these basic equations are written as a matrix equation:

(A.4)
$$\omega \left(\begin{array}{c} H\\ E\\ J \end{array}\right)=\left(\begin{array}{c} 0\;\frac{i}{\mu_{0}}\nabla \times \;0\\ -\frac{i}{\varepsilon_{0}}\nabla \times \;0\;\frac{i}{\varepsilon_{0}}\\ 0\;-i\varepsilon_{0}\omega_{pe}^{2}\;0 \end{array}\right)\left(\begin{array}{c} H\\ E\\ J \end{array}\right)$$

The above matrix equation can be rewritten as a Hamiltonian eigenvalue problem. Here, we set $x=\left(H,E,J\right)$ and Equation (A.4) is expressed as *ω* **Ax** = Bx. A is a diagonal matrix that can be represented as

(A.5)
$$A=\mathrm{diag}\left(\mu_{0},\varepsilon_{0},\frac{1}{\varepsilon_{0}\omega_{pe}^{2}}\right)$$

Combining Equations (A.4) and (A.5), matrix B can be obtained as follows

(A.6)
$$B=\left(\begin{array}{ccc} 0 & i\nabla \times & 0\\ -i\nabla \times & 0 & i\\ 0 & -i & 0 \end{array}\right)$$

It can be seen that both **A** and **B** are Hermitian, and **A** is positive definite. According to this property, we make $y=\sqrt{A}x$ . So, the eigenequation can be written as

(A.7)
$$\omega y={\left(\sqrt{A}\right)}^{-1}B{\left(\sqrt{A}\right)}^{-1}y$$

According to Equations (A.5) and (A.6), we can obtain

(A.8)
$${\left(\sqrt{A}\right)}^{-1}B{\left(\sqrt{A}\right)}^{-1}=\left(\begin{array}{ccc} 0 & i\frac{1}{\sqrt{\mu_{0}\varepsilon_{0}}}\nabla \times & 0\\ -i\frac{1}{\sqrt{\mu_{0}\varepsilon_{0}}}\nabla \times & 0 & i\omega_{pe}\\ 0 & -i\omega_{pe} & 0 \end{array}\right)$$

It is easy to recognize that ${\left(\sqrt{A}\right)}^{-1}B{\left(\sqrt{A}\right)}^{-1}$ is also Hermitian. Then, the eigenmodes **y**_*m*_ and **y**_*n*_ with the eigenfrequency of *ω*_*m*_ and *ω*_*n*_ satisfy the orthogonality condition,

(A.9)
$$\begin{array}{ll} \int dry_{m}\cdot y_{n} & =\int dr\left[\mu_{0}H_{m}^{*}\cdot H_{n}+\varepsilon_{0}\left(1-\frac{\omega_{pe}^{2}}{\omega_{m}\omega_{n}}\right)E_{m}^{*}\cdot E_{n}\right]=\delta_{mn} \end{array}$$

The normalization of the eigenfield of the Hamiltonian in Equation (A.9) corresponds to the energy density of the electromagnetic wave when collisionless plasma is considered.

## Appendix B: The lowest frequency bands calculated by tight-binding method

To obtain guided modes in the plasma cutoff region, we place the alumina columns into the plasma background and assume that electromagnetic waves hop between the columns. The hopping strength depends on the number of sites in a unit cell. So, the band structures can be captured by the tight-binding Hamiltonian

(A.10)
$$H=-t\underset{\left\langle i,j\right\rangle }{\sum }\left(c_{i}^{†}c_{j}+h.c.\right)$$

where $c_{i}^{†}$ and *c*_*j*_ are the creation and annihilation operators of the particle in the sites *i* and *j* which belong to different sub-lattices, and *t* is the hopping amplitude between lattice sites. The three hopping patterns are shown in Figure 5(a)–(c), respectively, and the hopping strengths are related to the distance between the sites which can be taken as $t_{0}=1,t_{1}=1.4,t_{0}^{\prime }=2$ . As an example, we show the Hamiltonian expression for lattice 2 by the tight-binding method as follows,

(A.11)
$$H=\left[\begin{array}{c} -2t_{0}\left(\cos k_{x}+\cos k_{y}\right)\;-t_{1}\\ -t_{1}\;-2t_{0}\left(\cos k_{x}+\cos k_{y}\right) \end{array}\right]$$

The band structures for the three lattices are shown in Figure 5(d). The energy band can be lowered by increasing the number of sites within a unit cell. This effect can be well used to design the passing band within plasma sheath.

We present the experimental results for lattices 1 and 2 in Figure 6. When a unit cell contains one column, the band structure is shown in Figure 6(a). An omnidirectional band gap appears between the first band and the second band. In the first band, there are eigenmodes below the plasma cutoff frequency as shown in red dots, which can support the transmission of electromagnetic waves. The transmission spectrum of electromagnetic waves at different discharge currents is shown in Figure 6(b). A bandgap appears in the range of 8–13.6 GHz when the plasma is not generated, which is consistent with the simulation result. The transmittance decreases with the increase of the discharge current below 5.04 GHz. However, guided modes appear in the range of 5.04–5.8 GHz compared to electromagnetic waves propagating in pure plasma. To further extend the scope of the guided modes, we design a composite lattice structure, which contains two alumina columns in a unit cell. The band structure of the composite lattice is shown in Figure 6(c), indicating that the first and second bands are opened and a wide bandgap appears. The number of eigenmodes below the plasma cutoff region is larger than that of the simple square lattice illustrated in Figure 6(a). Figure 6(d) shows the experimental results of this composite lattice. The guided modes (3.2–5.8 GHz) in the plasma cutoff region appear which is affected by the discharge currents. Guided modes below the plasma cutoff frequency originate from the coupling of evanescent waves between alumina columns.
